# *Bacillus anthracis* Aerosolization Associated with a Contaminated Mail Sorting Machine

**DOI:** 10.3201/eid0810.020356

**Published:** 2002-10

**Authors:** Peter M. Dull, Kathy E. Wilson, Bill Kournikakis, Ellen A.S. Whitney, Camille A. Boulet, Jim Y.W. Ho, Jim Ogston, Mel R. Spence, Megan M. MacKenzie, Maureen A. Phelan, Tanja Popovic, David Ashford

**Affiliations:** * Centers for Disease Control and Prevention, Atlanta, Georgia, USA; † Defence Research and Development Canada, Medicine Hat, Alberta, Canada

**Keywords:** *Bacillus anthracis*, anthrax, risk assessment, occupational exposure

## Abstract

On October 12, 2001, two envelopes containing *Bacillus anthracis* spores passed through a sorting machine in a postal facility in Washington, D.C. When anthrax infection was identified in postal workers 9 days later, the facility was closed. To determine if exposure to airborne *B. anthracis* spores continued to occur, we performed air sampling around the contaminated sorter. One CFU of *B. anthracis* was isolated from 990 L of air sampled before the machine was activated. Six CFUs were isolated during machine activation and processing of clean dummy mail. These data indicate that an employee working near this machine might inhale approximately 30 *B. anthracis-*containing particles during an 8-h work shift. What risk this may have represented to postal workers is not known, but the risk is approximately 20-fold less than estimates of sub-5 micron *B. anthracis*-containing particles routinely inhaled by asymptomatic, unvaccinated workers in a goat-hair mill.

In the fall of 2001, 22 cases of anthrax were confirmed or suspected throughout the eastern United States as a result of bioterrorist release of spores (1). Ten cases (seven inhalational and three cutaneous) occurred in workers at postal facilities in which envelopes contaminated with *Bacillus anthracis* spores were processed by high-speed sorting machines. Two contaminated envelopes passed through a sorting machine at the United States Postal Service Processing and Distribution Center in Washington, D.C. (Brentwood mail facility), on the morning of October 12. The facility was closed on October 21 after anthrax infection was diagnosed; four employees were eventually confirmed as having inhalational anthrax (2). During the 9-day period while the facility continued to operate, >2,000 employees processed >60 million pieces of mail. In addition to the primary aerosol to which workers may have been exposed, they may have had continual reexposure to *B. anthracis* spores during this period.

At the time of the anthrax release in the fall of 2001, little was known about the re-aerosolization potential of *B. anthracis* spores after initial dispersion. Much of what was known came from studies conducted by the United States and Canadian military biological defense programs, using surrogate biological agents dispersed outdoors at very high concentrations (10^5^–10^8^ agent-containing particles/m^2^). These studies showed that re-aerosolization can occur, but risk is considered to be low (3,4). No information was available to answer similar questions about re-aerosolization risk in an indoor occupational setting such as a postal facility.

To address the question of continued risk for workers, we conducted an expanded safety evaluation of the partially remediated mail facility. A stamp on one of the two contaminated envelopes indicated that it had passed through Delivery Bar Code Sorter machine no. 17 at the Brentwood mail facility. This sorter, which had been idle for >2 weeks, had been cleaned with 0.5% hypochlorite solution before our testing. We evaluated the potential health risk to workers near that sorter by activating it and conducting surface and air sampling.

## Methods

### Surface Sampling

Two surface sampling techniques were used. Rodac plates (65-mm tryptic soy agar [TSA] plates; Becton-Dickinson, Franklin Lakes, NJ) were pressed onto the surface being sampled. Immediately adjacent to the Rodac sampling site, swab sampling was performed with sterile rayon-tipped swabs moistened with a 0.5-mL solution of phosphate-buffered saline + 0.05% Tween 20 (PBS Tween). An approximately 100-cm^2^ area was swabbed with sequential vertical, horizontal, and diagonal strokes. The swabs were individually placed in sterile, dry 15-mL conical tubes. Sampling focused on areas in the machine (electrical components, beneath belts, etc.) that were unlikely to have been cleaned with the topical bleach application.

### Air Sampling

**Figure 1:**
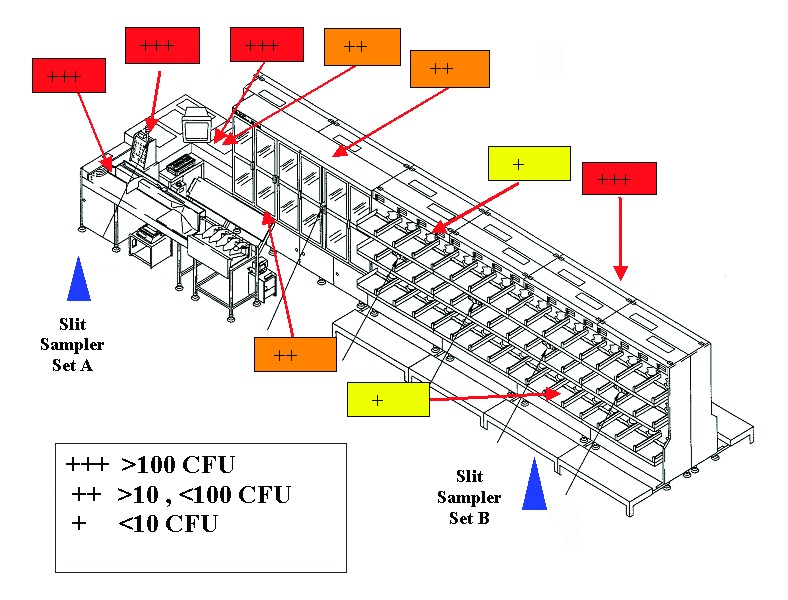
Sites and results of surface sampling for *Bacillus anthracis* with Rodac plates.

The ventilation system in the mail facility was turned off when the facility closed, and the system remained off during testing. We were unable to simulate the “blow-down” procedure (used to clean the sorter) during testing because the air compressors for the air hoses had lost power. Machine operators typically use high-pressure hoses several times a day to clean accumulated dust and debris between mail-sorting runs. Two banks of 10-slit samplers were placed on two postal trolleys (approximately 5 feet above the floor) and connected to a vacuum pump. The slit sampler intake ports were approximately 10 inches above the trolley. Each of the samplers was loaded with 150-mm TSA plates. Slit sampler set “A” (SSSA) was placed next to the operator’s station (at a location and height where workers would spend most of their time), and slit sampler set “B” (SSSB) was placed at the opposite end of the sorter (Figure 1). To measure the temporal patterns of re-aerosolization, the samplers operated sequentially; the intake port of slit sampler no.1 was opened, allowed to run for either 1 min (SSSA) or 2 min (SSSB), and then closed. Then slit sampler no.2 in the set was activated, and so on, until all 10 slit samplers in the set had been sequentially activated. The rate of air flow through each of the slit samplers was 33 L/min.

SSSB was activated and ran for 20 min while the sorter was turned off. SSSA was then activated and ran for 10 min. The plates were removed from all slit samplers and new plates were loaded. The SSSA and SSSB design characteristics determined the duration of sampling.

Both sets of slit samplers were activated simultaneously while the sorter was inactive. Approximately 1 min later, the sorter was started, and clean dummy mail was processed. After several false starts, continuous operation was achieved in approximately 2 min. The operation of the sorter was interrupted several times by jammed envelopes and quickly restarted each time, until the machine was turned off 1 min before the end of the 20-min sampling period. Postal officials reported that false starts, jamming, and restarting are common during routine operation of the machine.

As with the previous sampling, SSSA ran for 10 min and SSSB ran for 20 min. The plates were removed from the slit samplers and sealed in plastic bags. The bagged plates were taken out of the facility and the exteriors of the bags were decontaminated with 0.5% hypochlorite solution.

### Mask Filters

The sampling team was outfitted with Canadian military C4 respirators with C7 canisters. The mask was equipped with 37-mm glass fiber collection filters mounted on the inlet port of the C7 canister, so that the entire inspirational volume of the investigators was sampled. The masked team members were located near the sorter to provide additional point sampling of respirable aerosol during the experiment. Team members were stationed at different work sites along the sorter and elsewhere in the facility to serve as point detectors. All mask filters were worn for at least 2 h.

### Sample Handling and Processing

Environmental swabs and TSA plates from the Rodac plates and slit samplers were stored at 4°C until shipped and processed. All specimens were shipped at room temperature overnight to the Centers for Disease Control and Prevention. Swabs were placed in 1.5 mL PBS-Tween and vortexed for approximately 1 min. The solution was heat-shocked at 65°C for 30 min, and 100 µL was plated onto a sheep blood agar (SBA) plate. Rodac and slit sampler plates were incubated for approximately 12 h, and CFU were counted by visual inspection. All colonies suspected to be *Bacillus* spp. were subcultured on SBA plates. Identification and confirmatory testing of *B. anthracis* were done according to standard microbiologic procedures (5). The mask filters were removed in the facility and placed in sterile glass tubes. After transport, they were suspended in 3 mL of heart infusion broth and incubated at 35°C for 36 h, after which 10 µL of broth was plated onto SBA plates. The filters and remaining broth were heat-shocked at 65°C for 30 min, and 10 µL was plated onto SBA plates.

### Statistical Analysis

Numbers of colonies detected in air sampling before and after the machine was activated were assessed with a one-sided one-sample test for difference in rates from a binomial distribution by using StatExact 4 v. 4.0.1. (Cytel Software Corp., Cambridge, MA).

## Results

### Surface Sampling

Surface sampling was done by two methods, Rodac plates and premoistened swabs, to establish that the machine was still contaminated with viable *B. anthracis* spores. Ten Rodac plate samples and 10 swab samples were taken on the sorter surfaces. Both the Rodac plates and swabs yielded growth of CFUs that were too numerous to count at 7 of the 10 sites. Two additional Rodac plates were positive with low levels of contamination (1 and 3 CFUs); these locations were negative by the swab method (Figure 1).

### Air Sampling

**Figure 2:**
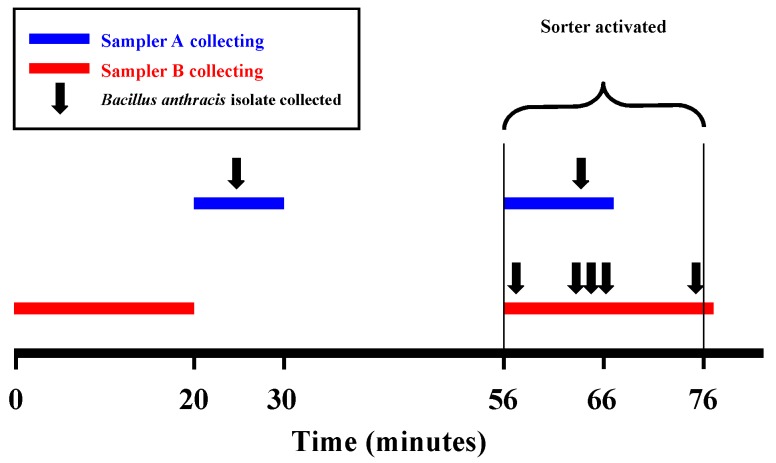
*Bacillus anthracis* air sampling: Slit Sampler Set A collected air samples for 10 minutes before and 10 min after the mail sorter was turned on. Slit Sampler Set B collected for 20 min during each period. Total air-flow rate, 33 L/min in both samplers.

A single colony of *B. anthracis* was identified on one of the SSSA plates (Figure 2) during 10 min of sampling before the sorter was activated. No *B. anthracis* was identified on any of the SSSB plates during the 20 min of sampling.

After the sorter was activated, SSSA and SSSB ran for 10 min and 20 min, respectively. A single colony of *B. anthracis* was identified during minute 10 of sampling by SSSA (Figure 2; Table). SSSB identified a single colony of *B. anthracis* at each of minutes 1, 5, 7, 8, and 19.

**Table:** Results of air sampling after Delivery Bar Code Sorter machine no. 17 was activated

Time (min)	1	2	3	4	5	6	7	8	9	10	11	12	13	14	15	16	17	18	19	20
Sampler A										+^a^										
Sampler B	+				+		+	+											+	

^a^+, positive for *Bacillus anthracis* CFU.

Five investigators wore mask filters while the sorting machine was inactive; four investigators changed to new mask filters while the sorting machine was active. All mask filters were negative by culture.

## Discussion

New questions have arisen as public health authorities have investigated and responded to the intentional release of *B. anthracis* in the United States. Studies by Canadian investigators with a sophisticated preparation of *Bacillus globigii* have shown that a contaminated envelope may, even unopened, cause a substantial primary aerosol event (6). In light of this new appreciation, we investigated whether, after a remote contamination event and initial decontamination, a Delivery Bar Code Sorter machine could be a continual source of aerosolized *B. anthracis* spores and, if so, whether the particle concentration in the air could be estimated.

Initial reports indicated that no specific remediation had yet been undertaken on the contaminated machine. Subsequently, we learned that the surface of the sorter implicated in processing the contaminated envelopes had been cleaned with 0.5% hypochlorite solution. We proceeded with testing because the expectation was that topical cleaning would provide only fractional decontamination of a contaminated machine. By focused sampling, we found that, despite topical cleaning, the sorter remained contaminated with *B. anthracis.* By either swab technique or Rodac plates, 9 of 10 sites on the machine were positive and 4 sites produced *B. anthracis* colonies that were too numerous to count.

Air sampling detected *B. anthracis* before and after the sorter was activated. Before the sorter was turned on, the samplers detected a single *B. anthracis-*containing particle (0.0010 agent-containing particles per liter of air [ACPL]). Six colonies of *B. anthracis* (0.0061 ACPL) were identified in the 990 L of air sampled after sorter activation. The difference between the number of *B. anthracis-*containing particles detected by the samples collected as background and those collected after the sorter was activated was not significant at the 0.05 level (p=0.06); however, analysis suggests a trend toward a significant increase.

Environmental surface sampling done shortly after the Brentwood mail facility was closed found widespread contamination of the facility with *B. anthracis* (7). Both aerosolization of *B. anthracis* spores and direct cross-contamination of surfaces were considered likely mechanisms for contamination. Approximately 30 h of air sampling with open-faced 37-mm mixed cellulose ester filters (0.8-µm pore size) was negative. The previous report of negative air sampling despite extensive testing suggests our detection of airborne *B. anthracis* while the sorter was inactive may have been spurious and possibly related to investigator activities while the experiment was being set up.

Based on these concentrations and assuming 100% sampler collection efficiency, the estimated number of *B. anthracis–*containing particles that a worker might inhale near this activated sorter can be calculated. If we assume a normal ventilation rate (10 L/min), during 8 h working around this partially cleaned, but still contaminated sorter, a worker might be expected to inhale approximately 30 *B. anthracis-*containing particles. This finding of very low-level airborne *B. anthracis* contamination is supported by the negative testing of the mask filters. If all the airborne particles are assumed to be of optimal size for inhalation, this estimate is approximately 100-fold less than the lower boundary of the 50% lethal dose estimates for inhalational anthrax in nonhuman primate studies (8). This number is also approximately 20-fold less than estimates of the number of routinely inhaled *B. anthracis*–containing particles from a 1960 study of asymptomatic, unvaccinated workers in a goat-hair mill in Pennsylvania (9). In that study, investigators calculated that, in an 8-h workday, workers inhaled >1,300 viable *B. anthracis-*containing particles, 510 of which were <5 μm in size. Thus, although detected in the Brentwood facility, airborne contamination was at a relatively low level.

The comparison of this type of exposure with nonhuman primate anthrax data and historical industrial anthrax data is problematic for several reasons. Our understanding of human infection risk at very low-dose *B. anthracis* exposures is limited, as illustrated by the death from inhalational anthrax of an elderly Connecticut woman for whom no exposure could be determined, despite extensive environmental testing of her home and areas she frequented (1). A well-known contributor to the rate of alveolar deposition of a bioaerosol is the particle size distribution; because the slit sampling method does not measure the aerodynamic particle size distribution, we were unable to measure this attribute. Finally, historical comparisons to goat-hair mill workers are limited by the unknown contributions of prior host immunity, incomplete surveillance, and the lack of additional environmental sampling data other than the study from Pennsylvania.

This study shows that a mail sorter may remain contaminated, as indicated by surface sampling, many days after processing *B. anthracis–*contaminated letters and despite topical bleach cleaning. In addition, even after processing >1.2 million subsequent letters, as this sorter did, aerosolized *B. anthracis–*containing particles can still be detected around a contaminated sorter when active, at a level likely increased over background levels. At the time of our study, the level of *B. anthracis–*containing particles around this contaminated sorter at Brentwood was low, but any level of aerosolized *B. anthracis* spores is undesirable in this occupational setting.

Further studies are essential to define the risks of inhalational anthrax in the settings of both primary and secondary aerosolization of *B. anthracis* spores. In anticipation of potential future *B. anthracis* exposures, re-aerosolization potential should be evaluated in other environments, such as an office setting. In addition, size stratification of the re-aerosolized portion of a primary release should be part of any testing, to give some guidance as to risk stratification for exposed persons. Finally, better understanding of human health risk of low-dose exposure of *B. anthracis* spores is critical to guide optimal public health response.
